# Development of peptone-based serum-free media to support Vero CCL-81 cell proliferation and optimize SARS-CoV2 viral production

**DOI:** 10.1016/j.heliyon.2024.e41077

**Published:** 2024-12-07

**Authors:** Suryo Kuncorojakti, Diena Delaiah, Ahmad Aswin, Yulianna Puspitasari, Yeni Damayanti, Helen Susilowati, Diyantoro, Iwan Sahrial Hamid, Mohammad Anam Al Arif, Suwarno, Watchareewan Rodprasert

**Affiliations:** a Division of Veterinary Anatomy, Department of Veterinary Science, Faculty of Veterinary Medicine, Universitas Airlangga, Surabaya, 60115, Indonesia; b Research Center for Vaccine Technology and Development, Institute of Tropical Disease, Universitas Airlangga, Surabaya, 60115, Indonesia; c Division of Veterinary Microbiology, Department of Veterinary Science, Faculty of Veterinary Medicine, Universitas Airlangga, Surabaya, 60115, Indonesia; d Department of Health Science, Faculty of Vocational Studies, Universitas Airlangga, Surabaya, 60115, Indonesia; e Division of Basic Veterinary Sciences, Department of Veterinary Science, Faculty of Veterinary Medicine, Universitas Airlangga, Surabaya, 60115, Indonesia; f Department of Animal Husbandry, Department of Veterinary Science, Faculty of Veterinary Medicine, Universitas Airlangga, Surabaya, 60115, Indonesia; g Veterinary Stem Cell and Bioengineering Innovation Center, Faculty of Veterinary Science, Chulalongkorn University, Bangkok, 10330, Thailand

**Keywords:** Serum-free media, Protein hydrolysate, Vaccine, Manufacture process, COVID-19

## Abstract

**Background:**

Developing an optimal media for Vero cell lines is crucial as it directly influences cell survival, proliferation, and virus production. The use of serum in cell culture raises safety concerns in biological production. The United States Food and Drug Administration (FDA) and the European Medicines Agency have implemented stricter regulations on the use of animal-derived components in commercial protein manufacturing to ensure patient safety.

**Objective:**

This study aims to develop new chemically defined media using peptone hydrolysate as a serum-free alternative.

**Methods:**

A rational experimental design was employed to screen and optimize potential serum substitutes. Six types of peptones were tested at varying concentrations to determine the top three candidates. These candidates were subsequently combined into five mixtures to identify the two most effective serum-free media for Vero CCL-81 cells. The evaluation criteria included morphology observation and proliferation assay. The two most promising serum-free formulations were further evaluated for their ability to support virus production and using TCID_50_ and absolute quantification by RT-qPCR.

**Results:**

The results of this study indicate that Mixture 4, consisting of 0.5 g/L Phytone™ Peptone, 0.5 g/L Difco™ Soytone, and 2 g/L Bacto™ Malt, significantly enhanced Vero CCL-81 cell proliferation and produced high viral titers. The developed medium demonstrated comparable performance to commercially available serum-free media.

**Conclusion:**

Peptone-based serum-free media provide a robust alternative for supporting Vero CCL-81 cell growth and are suitable for virus production.

## 1 Introduction

Vero cells, a continuous cell line derived from the kidney of African green monkeys, are commonly used to produce viral vaccines [1,2]. Although the addition of serum can promote Vero cell growth, its use has several significant drawbacks. One major concern is the potential risk of contamination by agents such as mycoplasma, viruses, and prions, which are associated with bovine spongiform encephalopathy (BSE) [3]. Moreover, the use of fetal bovine serum (FBS) in basic culture media raises ethical issues as it is sourced from the blood of bovine fetuses following the slaughter of pregnant cows. Other issues associated with FBS include batch-to-batch variability, inconsistent results, limited availability, complications in downstream processing due to its high protein concentration, and its high cost [4].

Due to the obstacles associated with serum use in cell cultures, developing chemically defined media (CDM), including serum-free media supplemented with peptone hydrolysate, offers a promising alternative [5,6]. However, no single CDM is universally suitable for all cell lines. Therefore, specific CDM formulations must be tailored to individual cell lines to optimize cell growth and productivity [3]. Chemically defined media are culture media containing precisely known and controlled components, such as inorganic and organic substances as well as protein additives such as epidermal growth factor (EGF), insulin, vitamins, amino acids, fatty acids, cholesterol, or peptone hydrolysates [7].

Peptone hydrolysates are protein products hydrolyzed through acid, alkali, enzymatic, or fermentation processes. They consist of undefined mixtures of low-molecular-weight substances such as amino acids, peptides, vitamins, and trace elements that are commonly used as supplements in serum-free media (SFM) to supply nutrients for cell cultures. SFM supplemented with hydrolysates is a cost-effective and practical alternative. The addition of hydrolysates has been demonstrated to promote cell growth and stimulate protein production, enhancing viral titers and protein quality in mammalian cells [8,9]. Although commercially available SFM, such as VP-SFM, are ready to use, they present challenges including high costs and limited availability. Therefore, developing a chemically defined medium containing serum-free media with peptone supplementation is a promising solution. By employing a rational experimental design, this study aims to develop a serum-free media formulation supplemented with peptone hydrolysates to enhance the proliferation of Vero CCL-81 cells specifically optimized for SARS-CoV-2 virus production.

## 2 Methods

### 2.1 Cell line

The cell lines used in this study were Vero E6 (ATCC® CRL-1586™) Passage 43 and Vero CCL-81 (ATCC® CCL-81™) Passage 28 (ATCC, VA, USA) obtained from the cell bank repository of the Research Center for Vaccine Technology and Development, Institute of Tropical Disease (RCVTD-ITD), Universitas Airlangga. The cells were cultured in serum-containing media (SCM) consisting of Minimum Essential Medium (MEM) (Gibco, New York, USA) with 10 % Fetal Bovine Serum (Gibco, New York, USA), 1 % Amphotericin (Gibco, New York, USA), and 1 % Penicillin-Streptomycin (Gibco, New York, USA) at 37 °C and 5 % CO_2_ in an incubator (Thermo, New York, USA).

### 2.2 Peptone hydrolysates treatment

Bacto™ TC Yeastole, Phytone™ Peptone, Difco™ Soytone, Bacto™ Yeast Extract, Bacto™ Malt, and Gibco Yeast Extract obtained from BD Peptone Starter Pack No. 3 (Gibco, New York, USA) were used in this study. Each hydrolysate was prepared at concentrations of 1 g/L, 3 g/L, 6 g/L and 9 g/L in phosphate-buffered saline (PBS) and sterilized through filtration using a syringe filter with a pore size of 0.2 μm (NEST, Jiangsu, China). The details of the peptone hydrolysate treatments are summarized in Table 1.

**Table 1:** Peptone concentrations for peptone hydrolysate treatments.

Peptone	Peptone Code	Peptone Concentration (g/L)	Basal Media (ml)	Peptone Stock Volume (ml)	Total Volume (ml)
Bacto™ TC Yeastolate	Y1	1	49.5	0.5	50
	Y2	3	48.5	1.5	50
	Y3	6	47.0	3.0	50
	Y4	9	45.5	4.5	50
Gibco Yeast Extract	BY1	1	49.5	0.5	50
	BY2	3	48.5	1.5	50
	BY3	6	47.0	3.0	50
	BY4	9	45.5	4.5	50
Bacto™ Yeast Extract	YE1	1	49.5	0.5	50
	YE2	3	48.5	1.5	50
	YE3	6	47.0	3.0	50
	YE4	9	45.5	4.5	50
Phytone™ Peptone	P1	1	49.5	0.5	50
	P2	3	48.5	1.5	50
	P3	6	47.0	3.0	50
	P4	9	45.5	4.5	50
Difco™ Soytone	D1	1	49.5	0.5	50
	D2	3	48.5	1.5	50
	D3	6	47.0	3.0	50
	D4	9	45.5	4.5	50
Bacto™ Malt	BM1	1	49.5	0.5	50
	BM2	3	48.5	1.5	50
	BM3	6	47.0	3.0	50
	BM4	9	45.5	4.5	50

### 2.3 Cell cultures

#### 2.3.1 Titration study

In the titration study, Vero CCL-81 cells at a density of 8.5 × 10^3^ cells/well were cultured in SCM in 96-well plates and incubated at 37 °C with 5 % CO_2_ for 24 h. Once the cells reached approximately 80 % confluency the following day, the media were removed and replaced with six types of media containing four different concentrations (Table 1). The cell cultures were maintained for three days, with media changes performed every 48 h. The objective of this titration study was to determine the best three types of peptones based on cell morphology and proliferation capacity, evaluated using the colorimetric MTT assay. Cell morphology was observed daily using an inverted microscope (Invitrogen™ EVOS™ XL Core Configured Cell Imager; Invitrogen, USA) at 100x magnification.

#### 2.3.2 Mixture study

In the mixture study, the best three types of peptones were selected and mixed at varying concentrations to produce five mixtures, as detailed in Table 2. This mixture study was aimed at determining the best two combinations of serum-free media (SFM) formulations for Vero CCL-81 cells. Firstly, Vero CCL-81 cells at a density of 8.5 × 10^3^ cells/well were cultured in SCM in 96-well plates and incubated at 37 °C with 5 % CO_2_ for 24 h. Once the cells reached approximately 80 % confluency, the media were removed and replaced with the treatment media. The cell cultures were maintained for four days, with media changes performed every 48 h. The best two formulas were determined based on morphological evaluation and proliferation assay.

**Table 2:** Formulas for the mixture study.

	Peptone 1 (g/L)	Peptone 2 (g/L)	Peptone 3 (g/L)	Basal Media (ml)	Total Volume (ml)
Mixture 1	2.0	0.5	0.5	48.50	50
Mixture 2	2.0	2.0	2.0	47.00	50
Mixture 3	0.5	2.0	0.5	48.50	50
Mixture 4	0.5	0.5	2.0	48.50	50
Mixture 5	0.5	0.5	0.5	49,25	50

#### 2.3.3 Media evaluation

Vero CCL-81 cells at a density of 0.7 × 10^6^ cells/cm^2^ were cultured in SCM in T25-flasks at 37 °C with 5 % CO_2_ for 24 h. The media were removed, replaced with four treatment media (two formulations from the mixture study and two commercial media, namely MEM 10 % and VP-SFM), and incubated at 37 °C with 5 % CO_2._ Once the cells reached approximately 80 % confluency, they were inoculated with the SARS-CoV-2 isolate 35 BP28 at 0.01 MOI. The incubation was continued at 37 °C with 5 % CO_2_. When the cytopathic effects (CPE) were observed, the virus was harvested and evaluated using TCID_50_ and RT-qPCR.

### 2.4 Tetrazolium-based colorimetric assay for cell proliferation

Cell proliferation assay was carried out every 24 h using 3-[4,5-dimethylthiazol-2-yl]-2,5 diphenyl tetrazolium bromide (MTT) at a concentration of 0.5 mg/mL (Sigma Aldrich, Darmstadt, Germany). The media were removed and washed using 100 μl of phosphate-buffered saline (PBS; Gibco, New York, USA). Subsequently, 100 μl of MTT working solution was added and the plates were incubated for 30 min at a temperature of 37° with 5 % CO_2_. After incubation, the MTT reagent was removed and added with 100 μl of DMSO to dissolve the formazan crystals (Sigma-Aldrich, Darmstadt, Germany). The plates were placed on an orbital shaker at 150 rpm and measured using a plate reader (Biobase, Shandong, China) at a wavelength of 570 nm to determine optical density (OD) values.

### 2.5 TCID_50_ assay

The TCID_50_ assay was conducted using harvested viruses from the previous study. Vero E6 cells at a density of 0.1 × 10^5^ cells/well were cultured in SCM in 96-well plates and incubated at 37 °C with 5 % CO_2_ for 24 h. Once the cells reached approximately 80 % confluency, the media were removed and replaced with 100 μl of serial virus dilutions ranging from 10^−1^ to 10^−12^ and incubated for 60 min. Subsequently, 100 μl of overlay media was added and further incubated at 37° with 5 % CO_2_. Cell morphology was observed daily until CPE were observed. On the termination day, the media were removed and washed using 100 μl of PBS. After removing the PBS, 100 μl of crystal violet was added, and the plates were incubated for 15 min at 37 °C with 5 % CO_2_. The crystal violet was removed and washed again using 100 μl of PBS. The viral titer was determined using the Reed and Muench formula.

### 2.6 Absolute quantification by RT-qPCR

Viral RNA was extracted from the harvested virus obtained from the previous study using the Promega ReliaPrep™ Viral TNA Miniprep System, Custom Kit (cat: AX4820). The extracted RNA was quantified using the Seegene PCR Allplex™ 2019-nCoV Assay Kit (cat: RP10250X). In this study, the viral quantification was based on the Ct value of the E gene of the SARS-CoV-2 virus. The absolute quantification (copy/μl) was determined using the synthetic standard control of SARS-CoV-2 control48 (B.1.1.529/BA.1)" (Twist Bioscience; 105204, CA, USA).

## 3 Data analysis

Data were presented in the form of graphs and images. The OD values, viral titers, and viral absolute quantities were statistically analyzed using SPSS Statistics Version 26 (n = 4). Parametric statistics were used if the data were normally distributed as determined by the Shapiro-Wilk test. To compare treatment groups, analysis of variance (ANOVA) was used, followed by Duncan's post hoc test. The significance was considered at a p-value of less than 0.05.

## 4 Results

### 4.1 Plant-based peptones promoting Vero CCL-81 cell growth

**Fig. 1:**
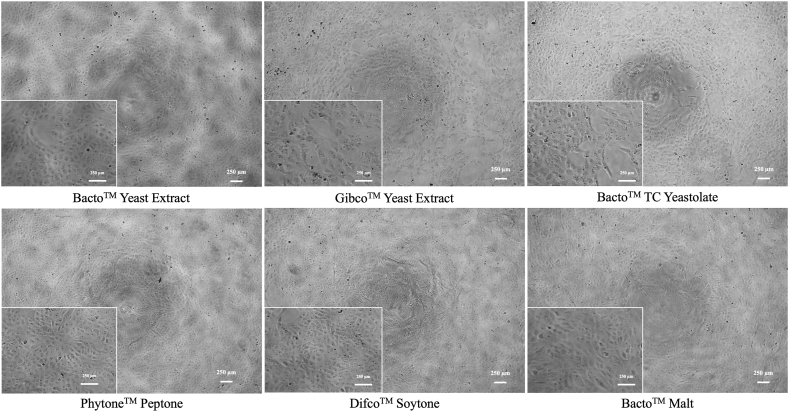
Morphological observation of Vero CCL-81 cells during the screening study on day 2.

Morphology observations indicated no significant changes in cell morphology across all treatment groups. The cells maintained an epithelioid and monolayer appearance, reaching more than 80 % confluency on the second day, as shown in Fig. 1. This result was supported by an increase in cell proliferation rate, as measured by the MTT assay.

**Fig. 2:**
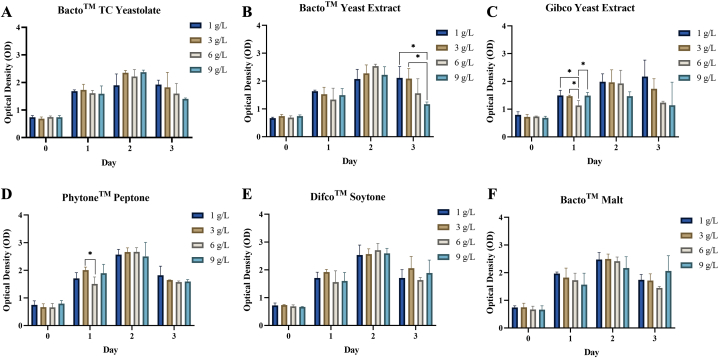
Proliferation assay during the titration study to determine the best-three peptones.

The proliferation assay results for Vero CCL-81 cells are presented in Fig. 2(A–F). The MTT assay revealed that peptone supplementation did not have a negative effect on Vero CCL-81 cells. All peptone types demonstrated a similar trend in promoting cell proliferation. The data indicated that a four-day incubation period was not optimal for Vero CCL-81 cells, with the highest OD value observed on day 3. The best three peptones were determined based on their OD values, namely Phytone™ Peptone, Difco™ Soytone, and Bacto™ Malt, all of which are plant-based peptones.

#### 4.1.1 Determination of the two best serum-free media formulas

**Fig. 3:**
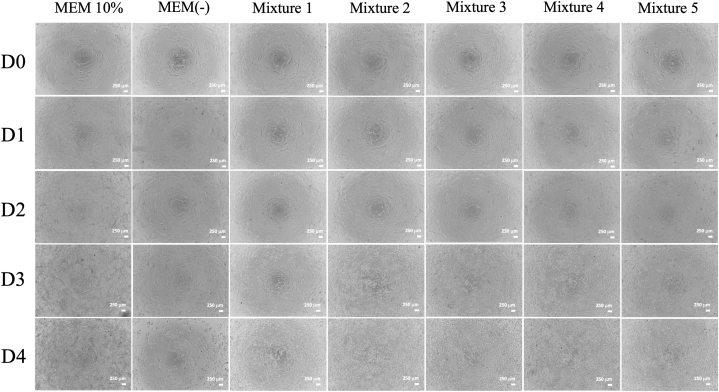
Microscopical evaluation to determine the normal morphology of Vero CCL-81 during the mixture study.

Microscopic observation of cell morphology showed no significant changes across all formulas. The cells maintained an epithelioid and monolayer appearance, reaching more than 80 % confluence on day 3, as shown in Fig. 3. These findings are consistent with the results of the proliferation assay, where the MTT assay demonstrated an increase in cell proliferation rate, as indicated by the OD values. In comparison, the serum-free media without any supplementation resulted in morphological changes, with cells appearing rounder, smaller, and unable to reach more than 80 % confluence on day 3.

**Fig. 4:**
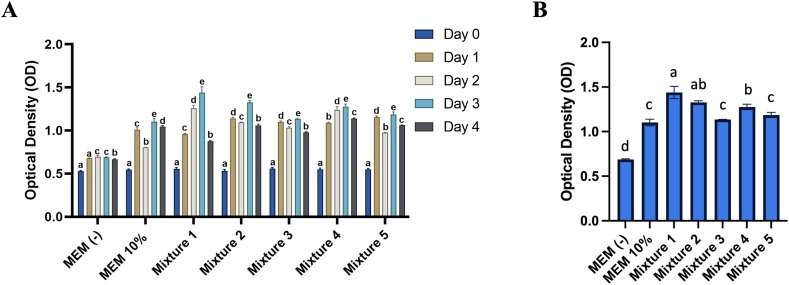
Proliferation assay to determine the best two formulas of peptone-based serum free media based on the evaluation within the treatment group (A) and between treatment groups on day 3.

According to the proliferation rate evaluation (Fig. 4A and B), Mixture 1 and Mixture 4 showed a consistent increase in optical density from day 0 to day 3. However, on day 4, a decrease in optical density was observed in all treatment groups. Mixture 2 and Mixture 3 showed a decrease in optical density on day 2 compared to the previous day, followed by an increase on day 3 and a decrease on day 4. Mixture 5 showed an increase in optical density on day 1, followed by a decrease on day 2, an increase on day 3, and a decrease on day 4. These results indicated inconsistency in the proliferation rate of Vero CCL-81 cells in these three groups. The two best peptone formulas were identified as Mixture 1, consisting of a combination of 2 g/L Phytone™ Peptone; 0.5 g/L Difco™ Soytone; and 0.5 g/L Bacto™ Malt, and Mixture 4, consisting of a combination of 0.5 g/L Phytone™ Peptone; 0.5 g/L Difco™ Soytone; and 2 g/L Bacto™ Malt.

#### 4.1.2 Peptone-based serum-free media promoting SARS-CoV2 titers

**Fig. 5:**
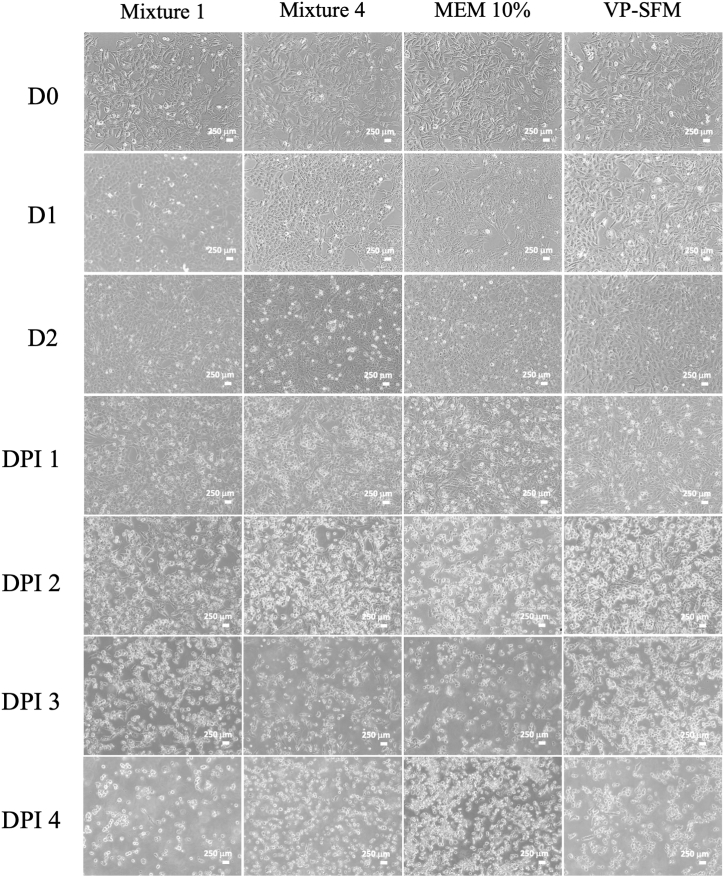
Microscopical evaluation of Vero CCL-81 cell before and after SARS-COV-2 virus inoculation.

Daily observations of cell morphology showed no changes, with the cells maintaining an epithelioid and monolayer appearance. Vero CCL-81 cells reached more than 80 % of confluence on day 2, as shown in Fig. 5. On day 0, the cells were inoculated with SARS-CoV-2 virus at 0.01 MOI. Following the virus inoculation, morphological observations were conducted daily to monitor morphological changes until CPE were observed. Starting from the second day post-inoculation, the cells appeared rounder and smaller. On the fourth day post-inoculation, the cells showed signs of death and detachment from the surface of the container.

**Fig. 6:**
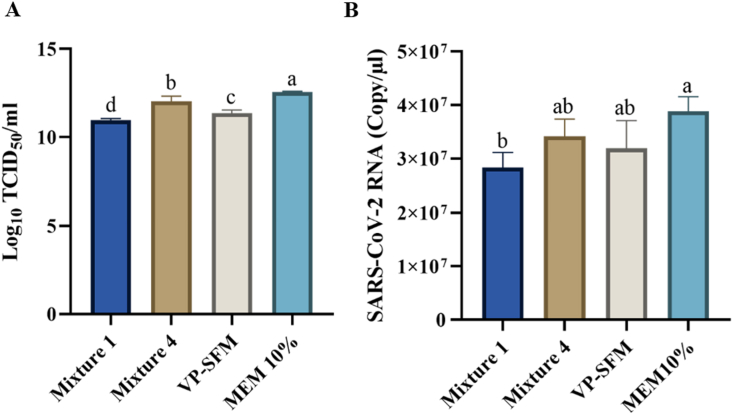
Virus quantification. (A) Average viral titer determination using TCID_50_; and (B) absolute quantification to determine viral RNA using RT-qPCR. Significant differences between groups are indicated by different notations (p < 0.05).

The virus production from the four treatment media groups, namely Mixture 1, Mixture 4, VP-SFM, and 10 % MEM, was quantified using the TCID_50_ method to determine the viral titer. The results were validated by absolute quantification through RT-qPCR to assess viral RNA levels. According to Fig. 6(A and B), both the TCID_50_ and absolute quantification using RT-qPCR results showed similar trends in virus quantification. The highest results were observed in the MEM 10 % group, followed by Mixture 4, VP-SFM, and Mixture 1.

## 5 Discussion

As demonstrated in previous studies [2,10], Vero cells exhibited epithelial-like morphology during the culture. However, the groups cultured in SFM without any supplementation failed to maintain normal cell morphology, with the cells appearing rounder and smaller. Morphological changes can occur due to the lack of nutrients in the SFM group, such as glutamine and galactose, which may promote apoptosis [11]. Round-shaped apoptosis is a form of programmed cell death. During the early stages of apoptosis, microtubules and intermediate filaments are disorganized and the actin cytoskeleton aids in cell reorganization [12]. Supplementation with FBS provides essential nutrients for cell survival, such as plasma proteins, peptides, fats, growth factors, hormones, and inorganic substances that may influence their biological behavior and stress response [13,14]. In apical papilla cells, the absence of FBS has been shown to reduce proliferation and alter biological characteristics [15]. Due to batch-to-batch variability, adverse effects in clinical use, and ethical concerns, the use of FBS in manufacturing processes has become increasingly discouraged [14,16]. Numerous alternatives have been developed to replace the use of serum. One potential approach is the hydrolysate peptone supplementation [17]. Hydrolysate peptones contain amino acids, vitamins, trace elements, and specific peptides that may resemble growth factors or survival factors [3], along with glutamine, which might protect cells from apoptosis [9,11]. In this study, plant-based peptone was chosen as an alternative supplement due to the animal-free composition, which alleviate challenges related to bio-product release licensure requirements by the regulator. In the past, concerns about batch-to-batch variation limited the use of peptones. Currently, the standardization and manufacturing processes for protein hydrolysates are well-established. A previous study showed that Vero cell proliferation rates were not affected by peptone supplementation from different production batches, suggesting that the inter-batch variation can be overcome by using commercially standardized peptones [18].

The MTT assay measures the metabolic activity of live cell mitochondria to convert the yellow color of tetrazolium salt (3-(4,5-dimethylthiazol-2-yl)-2,5-diphenyltetrazolium bromide) into a purple formazan crystal product [19]. Since only live cells with intact mitochondria and cell membranes can catalyze this reaction, MTT has been frequently used to assess cell proliferation [20,21]. This method is based on the mitochondrial metabolic rate, which can indirectly reflect the number of viable cells [21].

According to the titration study, varying concentrations of each peptone were used to determine the proliferation rate of Vero CCL-81 cells. The results of this study indicate that the varying concentrations did not affect cell growth. A similar pattern in the cell proliferation assay was observed, where the proliferation rate increased daily until day 2, followed by a decrease on day 3. This phenomenon may be attributed to the cells fulfilling the surface of the entire container, inhibiting cell proliferation due to the lack of attachment sites on the well's bottom surface. According to Nelson, increased cell-to-cell contact in adherent cells prevents growth factor and integrin-mediated stimuli for cell division and proliferation. This density-dependent inhibition occurs at a saturation density specific to the cell culture [22].

The MTT results of this study identified the best three peptones as Phytone™ Peptone, Difco™ Soytone, and Bacto™ Malt. These peptones were subsequently used in the mixture study to determine the best two media formulations. The results of the mixture study showed that the highest cell proliferation was observed in Mixture 1 and Mixture 4. Cell proliferation is influenced by the nutrients present in the culture media, which provide all necessary nutrients for cell growth, proliferation, and metabolism [19]. The required nutrients for cell culture generally include peptides, glucose, inorganic salts, and carbohydrates. These nutrients are present in peptone hydrolysates, making them suitable for supplementation in serum-free media. A previous study by Spearman et al. (2016) [23] indicated that peptides containing arginine and lysine play important roles in the growth and proliferation of CHO cells. Peptides can function as signaling molecules and regulate various cellular processes, including proliferation, differentiation, and cell apoptosis. In addition, peptides can serve as growth factors and anti-apoptotic agents [24,25]. Protein hydrolysates not only offer a supply of useable amino acids, but also contain peptides that can have specific effects, such as mimicking growth factors or survival factors [26].

The comparable virus yield in this study may be associated with the proliferation rate of the cells. Peptone-based serum-free media yielded comparable results in terms of the proliferation rate of Vero CCL-81 cells. Cell proliferation and metabolism play a crucial role in virus production as living cells actively engage in growth, division, and proliferation. When a virus infects and hijack the cell, it takes over the cell's replication mechanisms and utilizes them to produce viral RNA proteins. Viruses also exploit the metabolic resources of the cell. This aligns with the study conducted by Girdhar et al. (2021) [27], which stated that viral regulation of host metabolism is a critical component of virus replication. The interaction between cell proliferation mechanisms and metabolism, influenced by viral infection, can contribute to virus production outcomes, leading to increased viral titers. This is consistent with the research by Ho et al. (2021) [9], which indicated that peptone hydrolysates can enhance CHO cell proliferation and act as a protein production stimulant to increase viral titers and product quality. In this study, the two most promising peptone-supplemented serum-free media formulations yielded different levels of virus production. Although there is no clear explanation from previous studies, it can be hypothesized that the certain stress condition such as high-density condition of Vero cells can secrete antiviral properties (interferon gamma). This is supported by a previous transcriptomic study on Vero cells, which revealed that under stress conditions, there is an upregulation of the interferon gamma response and a downregulation of MYC targets (variants 1 and 2), E2F target pathways, and most importantly, the epithelial-mesenchymal transition pathway [28].

## 6 Conclusion

The use of serum-free media based on peptone hydrolysates from Mixture 4, consisting of a combination of 0.5 g/L Phytone™ Peptone; 0.5 g/L Difco™ Soytone; and 2 g/L Bacto™ Malt, has been shown to enhance Vero CCL-81 cell proliferation while maintaining normal morphology. This formula can serve as an alternative medium for the SARS-COV-2 propagation, yielding viral titers comparable to those obtained using commercially available serum-free media. These findings provide baseline data for the development of cell culture-based vaccine production for SARS-COV-2. Nevertheless, this study has limitations, as all experiments were conducted in a 2D culture system. Further research needs to be conducted in a larger scale using a 3D culture system to validate these results on SARS-COV-2 titers to support the translation into large-scale vaccine production.

## CRediT authorship contribution statement

**Suryo Kuncorojakti:** Writing – review & editing, Visualization, Validation, Supervision, Methodology, Funding acquisition, Formal analysis, Data curation, Conceptualization. **Diena Delaiah:** Writing – original draft, Visualization, Methodology, Investigation, Formal analysis, Data curation. **Ahmad Aswin:** Writing – review & editing, Visualization, Methodology, Investigation. **Yulianna Puspitasari:** Writing – review & editing, Validation, Supervision, Project administration, Methodology. **Yeni Damayanti:** Writing – review & editing, Validation, Supervision, Project administration. **Helen Susilowati:** Writing – review & editing, Validation, Project administration, Investigation. **Diyantoro:** Writing – review & editing, Project administration, Methodology, Investigation, Writing – review & editing, Project administration, Methodology, Investigation. **Iwan Sahrial Hamid:** Writing – review & editing, Validation, Supervision. **Mohammad Anam Al Arif:** Writing – review & editing, Validation, Supervision, Formal analysis. **Suwarno:** Writing – review & editing, Validation, Supervision, Writing – review & editing, Validation, Supervision. **Watchareewan Rodprasert:** Writing – review & editing, Visualization, Supervision, Methodology, Conceptualization.

## Funding Statement

This study was supported by Airlangga Research Fund (Penelitian Dasar Unggulan/PDU) Grant Number 1512/UN3.FKH/PT.01.03/2024.

## Ethical approval

Not required.

## Ethics statement

Not required.

## Data availability

The datasets used and/or analyzed in this study are available upon request to the corresponding author.

## Declaration of competing interest

The authors declare the following financial interests/personal relationships which may be considered as potential competing interests:Suryo Kuncorojakti reports financial support was provided by Faculty of Veterinary Medicine Universitas Airlangga. If there are other authors, they declare that they have no known competing financial interests or personal relationships that could have appeared to influence the work reported in this paper.
